# The infantile myofibromatosis NOTCH3 L1519P mutation leads to hyperactivated ligand-independent Notch signaling and increased PDGFRB expression

**DOI:** 10.1242/dmm.046300

**Published:** 2021-02-24

**Authors:** Dan Wu, Sailan Wang, Daniel V. Oliveira, Francesca Del Gaudio, Michael Vanlandewijck, Thibaud Lebouvier, Christer Betsholtz, Jian Zhao, ShaoBo Jin, Urban Lendahl, Helena Karlström

**Affiliations:** 1 Department of Neurobiology, Care Science and Society, Karolinska Institutet, 171 77 Stockholm, Sweden; 2 Department of Obstetrics and Gynecology, Women's Hospital of Nanjing Medical University, Nanjing Maternity and Child Health Care Hospital, Nanjing 211166, China; 3 Department of Medicine, Karolinska Institutet, 171 77 Stockholm, Sweden; 4 Department of Cell and Molecular Biology, Karolinska Institutet, 171 77 Stockholm, Sweden; 5 Department of Medicine, Karolinska Institutet, 141 86 Huddinge, Sweden; 6 Integrated Cardio Metabolic Center (ICMC), Karolinska Institutet, 141 86 Huddinge, Sweden; 7 Department of Immunology, Genetics and Pathology, Uppsala University, 751 85 Uppsala, Sweden; 8 Inserm U1171, University of Lille, CHU, Memory Center, Distalz, F-59000 Lille, France; 9 Department of Oncology-Pathology, Karolinska Institutet, 171 77 Stockholm, Sweden

**Keywords:** Infantile myofibromatosis, Notch, PDGF, Fibroblast

## Abstract

Infantile myofibromatosis (IMF) is a benign tumor form characterized by the development of nonmetastatic tumors in skin, bone, muscle and sometimes viscera. Autosomal-dominant forms of IMF are caused by mutations in the *PDGFRB* gene, but a family carrying a L1519P mutation in the *NOTCH3* gene has also recently been identified. In this study, we address the molecular consequences of the NOTCH3^L1519P^ mutation and the relationship between Notch and PDGFRB signaling in IMF. The NOTCH3^L1519P^ receptor generates enhanced downstream signaling in a ligand-independent manner. Despite the enhanced signaling, the NOTCH3^L1519P^ receptor is absent from the cell surface and instead accumulates in the endoplasmic reticulum. Furthermore, the localization of the NOTCH3^L1519P^ receptor in the bipartite, heterodimeric state is altered, combined with avid secretion of the mutated extracellular domain from the cell. Chloroquine treatment strongly reduces the amount of secreted NOTCH3^L1519P^ extracellular domain and decreases signaling. Finally, NOTCH3^L1519P^ upregulates PDGFRB expression in fibroblasts, supporting a functional link between Notch and PDGF dysregulation in IMF. Collectively, our data define a NOTCH3–PDGFRB axis in IMF, in which an IMF-mutated NOTCH3 receptor elevates PDGFRB expression. The functional characterization of a ligand-independent gain-of-function NOTCH3 mutation is important for Notch therapy considerations for IMF, including strategies aimed at altering lysosome function.

## Abstract (precis)

**Summary:** Analysis of the L1519P mutation in NOTCH3 and the relationship between Notch and PDGF signaling in infantile myofibromatosis revealed that NOTCH3^L1519P^ generates hyperactivated, ligand-independent Notch signaling, which acts epistatically over PDGF signaling.

## INTRODUCTION

Infantile myofibromatosis (IMF, MIM 228550) patients suffer from nonmetastatic tumors that develop in skin, bone, muscle and viscera (Chung and Enzinger, 1981). IMF has an estimated incidence of 1:150,000-400,000 live births and was first described as a distinct disease entity in 1954 (Stout, 1954). The term ‘infantile myofibromatosis’ was coined in 1981 (Chung and Enzinger, 1981). IMF tumors, which most often occur in children, are generally benign and sometimes regress, although visceral tumors can be lethal (Schurr and Moulsdale, 2008). Histological findings comprise fascicles of spindle-shaped cells separated by collagen fibers surrounding a central vascular area with features of hemangioperiocytoma (Levine et al., 2012).

IMF can occur in both autosomal-recessive and -dominant forms, suggesting complex underlying genetics. For the autosomal-dominant forms, two mutations in the *PDGFRB* gene (MIM 173410) have been identified (Cheung et al., 2013; Martignetti et al., 2013). One report identified *PDGFRB* c.1681C>T, p.Arg561Cys (R561C) and c.1978C>A, p.Pro660Thr (P660T) mutations in eight families (Martignetti et al., 2013), while another study identified 11 affected patients carrying the R561C mutation (Cheung et al., 2013). The *PDGFRB* mutations lead to ligand-independent receptor activation (Arts et al., 2017) and are likely gain-of-function mutations, as they are sensitive to kinase inhibitors (Mudry et al., 2017). Elevated expression of PDGF ligands and receptors has also been observed in pediatric fibromatoses and myofibromatosis (Gibson et al., 2007), further supporting the notion that elevated PDGF signaling can cause IMF. PDGF signaling is initiated by PDGF ligands, which cause PDGF receptor dimerization, leading to receptor autophosphorylation and phosphorylation of downstream target proteins; for example, in the MAPK, PI3K and JAK/STAT pathways (Heldin, 2013). PDGFRB, and its main ligand, PDGF-B, are highly evolutionarily conserved (Hoch and Soriano, 2003). During development, PDGF signaling is paramount for the recruitment of pericytes during blood vessel formation, and complete knockout of either the *Pdgfb* or *Pdgfrb* gene in mice leads to perinatal death (Leveen et al., 1994; Soriano, 1994).

**Fig. 1.**
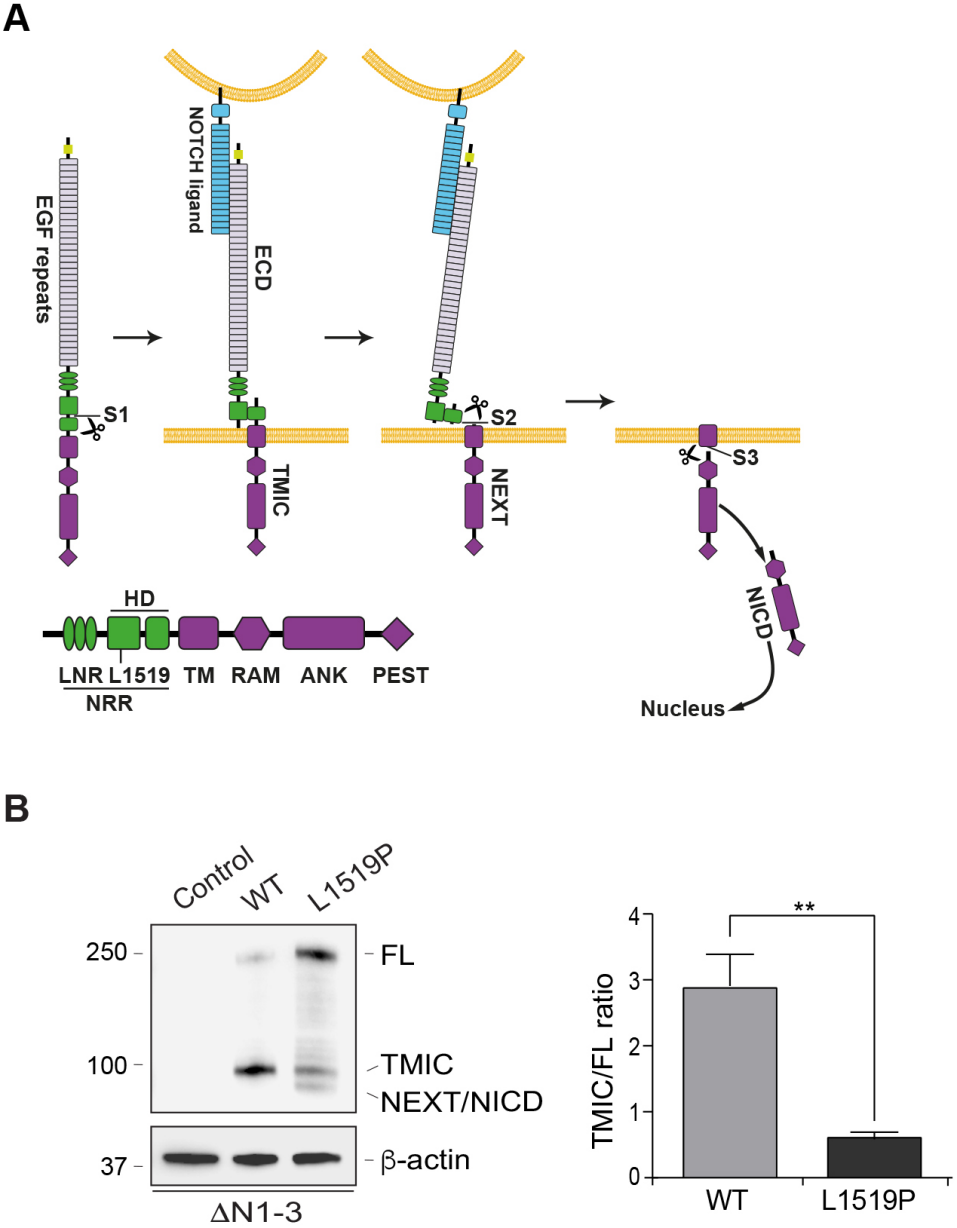
**Aberrant processing of NOTCH3^L1519P^.** (A) Schematic overview of Notch proteolytic processing. (B) Expression of the full-length (FL), transmembrane intracellular domain (TMIC) and Notch extracellular truncated (NEXT)/Notch intracellular domain (NICD) forms was analyzed by western blotting of cell extracts from HEK 293T ΔN1-3 cells (Control) or HEK 293T ΔN1-3 cells expressing wild-type (WT) and NOTCH3^L1519P^ (L1519). β-actin levels were used as a loading control (*n*=3). Relative levels of TMIC/FL were quantified by ImageJ from three experiments and analyzed by paired Student's *t*-test, **P<0.01. ECD, extracellular domain; EGF, epidermal growth factor; NRR, negative regulatory region.

Recently, mutations have also been identified in the *NOTCH3* gene in IMF patients. Nine affected individuals in an IMF family carried a heterozygous c.4556 C>T mutation in *NOTCH3*, whereas seven unaffected family members did not harbor the mutation (Martignetti et al., 2013; for review, see Lee, 2013). The c.4556 C>T mutation results in a leucine to proline transition at position 1519 in the NOTCH3 receptor (L1519P), a site located in the so-called heterodimerization domain of the receptor (Fig. 1A). Notch receptors (NOTCH1-4) are large transmembrane receptors present at the cell surface as dipartite (heterodimeric) proteins after proteolytic cleavage in the Golgi compartment. This first cleavage, called Site 1 (S1) cleavage, is executed by furin-like convertase, generating the extracellular domain (ECD) and the transmembrane intracellular domain (TMIC) (Fig. 1A). The ECD and TMIC moieties are held together via the two halves of the heterodimerization domain, which is split up by the S1 cleavage (Fig. 1A). At the cell surface, the receptor interacts with Notch ligands (of the Dll and Jagged type) presented at a juxtaposed cell, and the ligand-receptor interaction results in a second proteolytic (S2) cleavage conducted by ADAM metalloproteinases (Fig. 1A). The S2 cleavage occurs when a ‘hinge’ region in the so-called negative regulatory region (NRR; which encompasses the heterodimerization domain and the three LNR repeats; Fig. 1A) opens up in response to a pulling force from the ligand, exposing the S2 cleavage site. S2 cleavage yields the Notch extracellular truncated (NEXT) form of the receptor from the TMIC, and NEXT is directly processed by the γ-secretase complex (S3 cleavage) to produce the intracellular domain (ICD). The S3 cleavage is intramembranous and occurs at the cell surface or in endosomes (Fig. 1A) (Siebel and Lendahl, 2017). The S3 cleavage releases the Notch ICD, which acts as a transactivating factor after binding to the DNA-binding CSL (RBP-j) protein. In the absence of Notch activation, CSL acts as a transcriptional repressor but converts to become an activator upon binding of Notch ICD and a third protein, MAML, into a trimeric Notch ICD/MAML/CSL complex. Notch signaling is, like PDGF signaling, used in many organs and important for organ development as well as tissue homeostasis (Siebel and Lendahl, 2017). In addition to being mutated in IMF, mutations in *NOTCH3* cause the stroke and dementia syndrome cerebral autosomal-dominant arteriopathy with subcortical infarcts and leukoencephalopathy (CADASIL; OMIM 125310), which is characterized by white matter lesions, lacunar ischemic infarcts and degeneration of vascular smooth muscle cells in the brain vasculature (Coupland et al., 2018).

In this study, we analyze the molecular consequences of the L1519P IMF mutation in the NOTCH3 protein and the relationship between Notch and PDGF signaling in IMF. We find that NOTCH3^L1519P^ generates hyperactivated Notch signaling, despite the fact that it does not appear at the cell surface. The dimeric form of NOTCH3^L1519P^ is relocalized to the perinuclear area, and the ECD is avidly exported from the cell to the surrounding medium, indicating aberrant intracellular routing. We also show that NOTCH3^L1519P^ exacerbates *PDGFRB* expression, supporting the notion of a NOTCH3–PDGFRB axis, in which Notch is epistatic over PDGF signaling. The notion of a ligand-independent hyperactivated Notch receptor in IMF has important implications for Notch therapy development.

## RESULTS

### NOTCH3^L1519P^ exhibits an imbalance between the full-length and TMIC forms

To explore the molecular consequences of the *NOTCH3^L1519P^* mutation, we engineered cell lines in which the wild-type or L1519P forms of *NOTCH3* were introduced into human embryonic kidney (HEK) 293T cells from which we had first ablated the *NOTCH1*, *NOTCH2* and *NOTCH3* genes by CRISPR/Cas9 (HEK 293T ΔN1-3 cell line), to rid the cells of endogenous Notch signaling and thus eliminate the potential risk of antibody cross-reactivity in the cellular localization assays described below (Fig. S1A). The wild-type or L1519P forms of *NOTCH3* were stably integrated into the AAVS1 locus in the HEK 293T ΔN1-3 cell line, which is tetracycline regulated, thus allowing the levels of NOTCH3 receptor expression to be regulated by doxycycline stimulation (Fig. S1B). HEK 293T ΔN1-3 cell lines expressing similar amounts of wild-type NOTCH3 or NOTCH3^L1519P^ in the presence of doxycycline were selected (Fig. S1C). In the ‘Notch-off’ situation, i.e. without ligand stimulation, the wild-type NOTCH3 receptor appeared as a combination of a band representing the full-length (unprocessed) form (250 kDa) and the S1-processed TMIC form (90 kDa), as expected (Fig. 1B). In contrast, the NOTCH3^L1519P^ receptor appeared predominantly as the unprocessed full-length form, with only minor amounts of the TMIC and NEXT/ICD forms (NEXT and ICD migrate very closely together and cannot be separated under the western blot conditions used in this experiment) (Fig. 1B). In all, this suggests an imbalance between the full-length and processed forms (TMIC and NEXT/ICD) of NOTCH3^L1519P^.

### Aberrant intracellular routing of NOTCH3^L1519P^

**Fig. 2.**
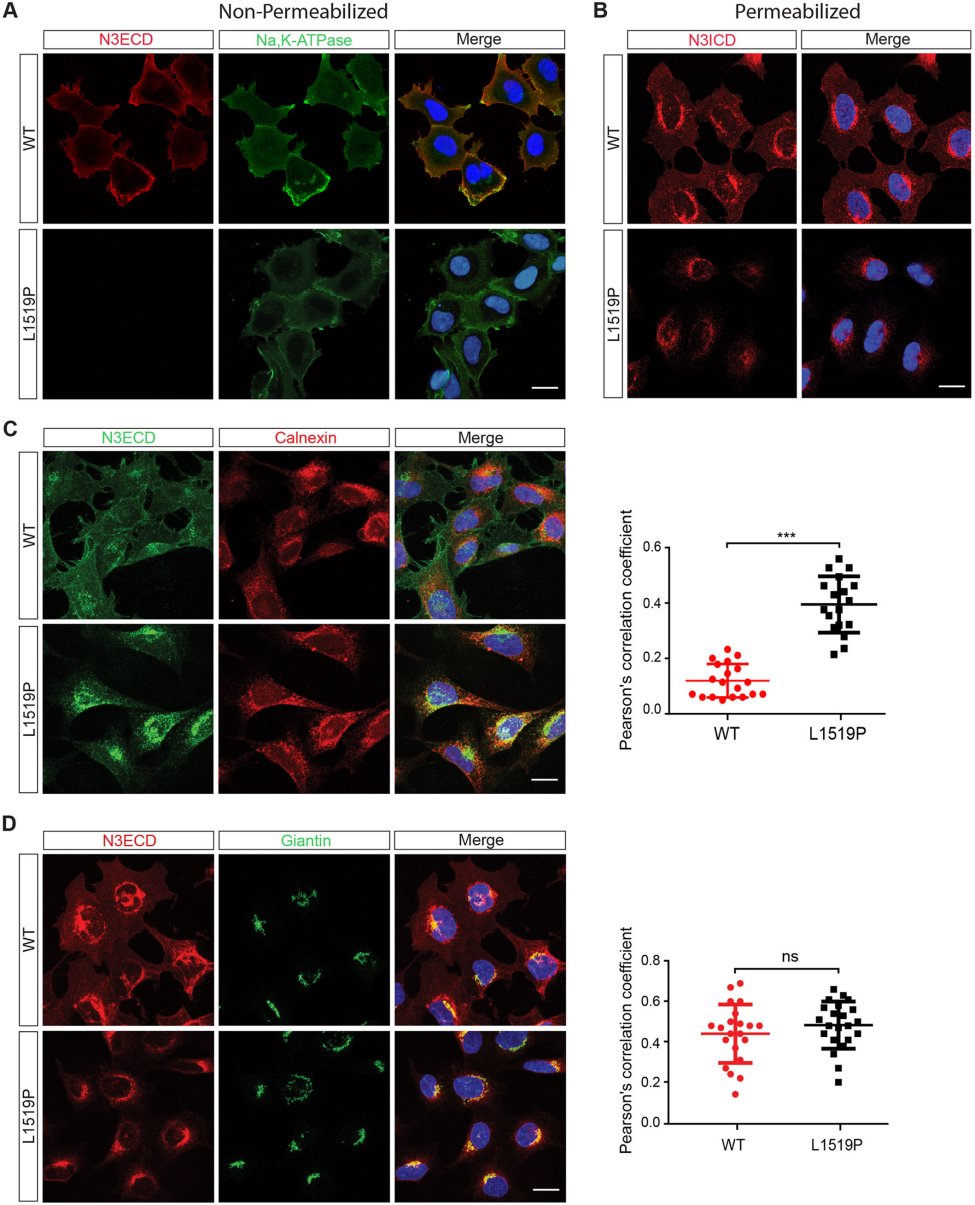
**Lack of cell surface expression and increased retention of NOTCH3^L1519P^ in the endoplasmic reticulum (ER).** (A,B) Confocal images of wild-type and NOTCH ^L1519P^-expressing cells without (A) and with (B) permeabilization. Na,K-ATPase was used as a membrane marker. (C) Immunocytochemistry for NOTCH3 ECD (green) and the ER marker calnexin (red) from permeabilized cells; the plot shows the Pearson's correlation coefficients for the colocalization analysis. (D) Immunocytochemistry for NOTCH3 ECD (red) and the Golgi marker giantin (green); the graph shows the Pearson's correlation coefficient analysis (*n*=3). Significance was calculated using unpaired Student's *t*-test, ***P<0.001; ns, not significant. Scale bars: 20 µm.

The imbalance between the full-length and processed forms of NOTCH3^L1519P^ may indicate a problem with intracellular routing, in S1 processing, which occurs in the Golgi compartment (Siebel and Lendahl, 2017), or in the stability of the heterodimer formed by the TMIC and ECD (Fig. 1A). To gain insights into the routing of the receptor from the endoplasmic reticulum (ER) to the cell surface, we assessed whether NOTCH3^L1519P^ routed to the cell surface. Immunostaining for the NOTCH3 ECD on intact, non-permeabilized cells revealed the presence of wild-type NOTCH3 at the cell surface, whereas, in contrast, NOTCH3^L1519P^ was undetectable at the cell surface, which was visualized by Na,K-ATPase staining (Fig. 2A). As a control for the amounts of wild-type and L1519P NOTCH3 protein, immunostaining for NOTCH3 ICD after permeabilization of the cells revealed ample amounts of NOTCH3 protein in both wild-type and mutant cells (Fig. 2B). To corroborate these data, a combined image streaming and microscopy analysis for NOTCH3 cell surface-immunostained cells confirmed that no or very little NOTCH3^L1519P^ was present at the cell surface (Fig. S2).

To gain further insights into the intracellular routing of NOTCH3^L1519P^, we explored its localization in the ER and Golgi compartments. Co-immunostaining with an antibody against the ECD and calnexin, a marker for the ER, revealed a more extensive colocalization with ER for NOTCH3^L1519P^ compared to wild-type NOTCH3 in permeabilized cells (Fig. 2C). Quantification of the colocalization using Pearson's correlation coefficient confirmed the increased accumulation of NOTCH3^L1519P^ in the ER (Fig. 2C). Co-immunostaining for the NOTCH3 ECD and the Golgi compartment, using giantin (GOLGB1) as a Golgi marker, showed no statistical difference between the wild-type and NOTCH3^L1519P^ protein (Fig. 2D). In sum, these experiments suggest that NOTCH3^L1519P^ is absent from the cell surface but shows increased retention in the ER.

### NOTCH3^L1519P^ undergoes enhanced and ligand-independent S2 cleavage

**Fig. 3.**
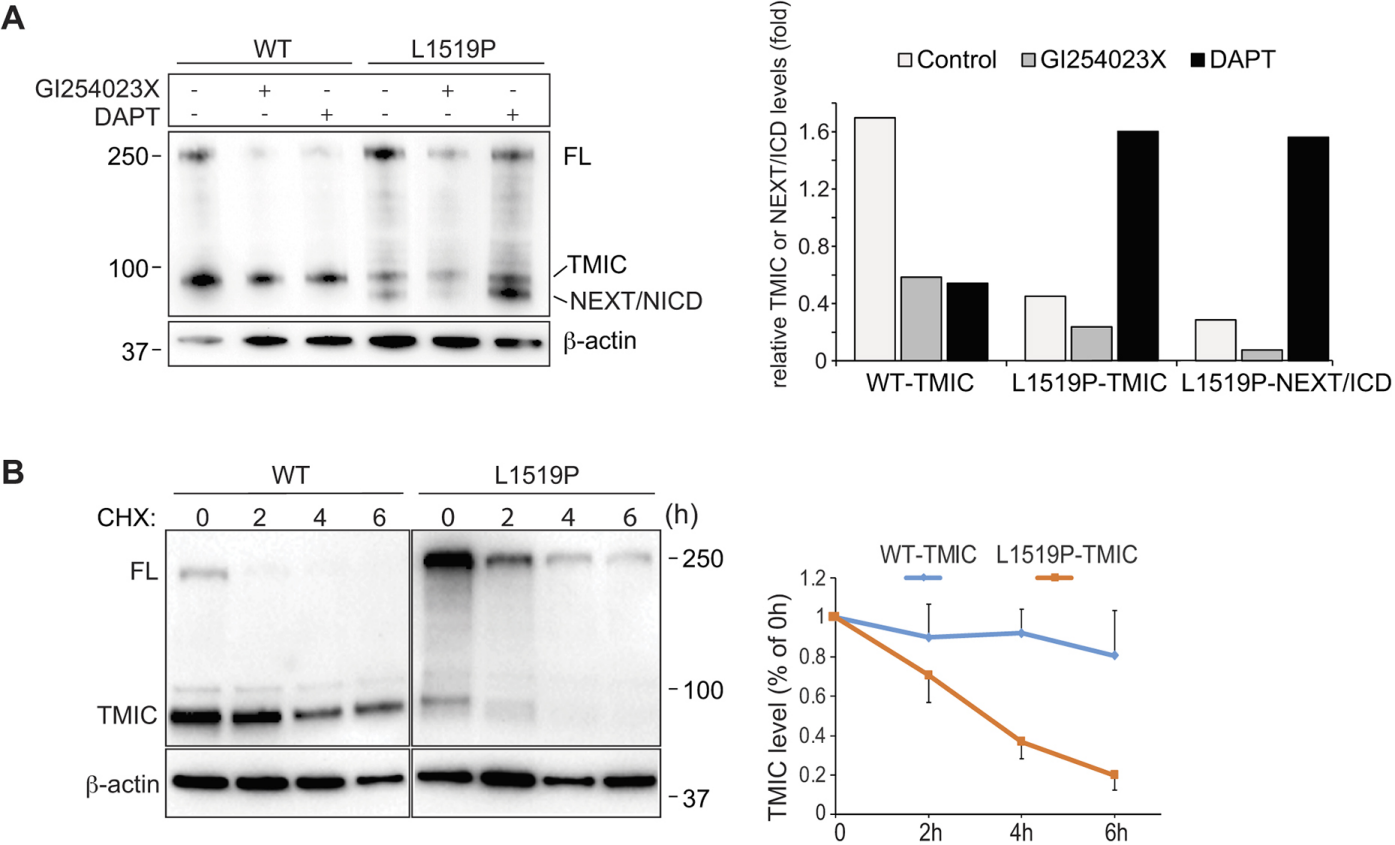
**NOTCH3^L1519P^ undergoes enhanced and ligand-independent S2 cleavage.** (A) Western blot analysis of TMIC and NEXT/NICD fragments in the presence of the S2 inhibitor GI254023X or the γ-secretase inhibitor DAPT. (B) Treatment of the wild-type and NOTCH3^L1519P^-expressing cells with cycloheximide at different time points, as indicated. The graph shows densitometric quantification of TMIC using ImageJ (*n*=3). Data are from three independent experiments.

To further investigate the basis for the aberrant receptor routing and altered TMIC levels, we explored whether S2 processing was affected in NOTCH3^L1519P^. In the Notch-off situation, i.e. when cells were not ligand stimulated, there was, as expected, a build-up of TMIC from the wild-type NOTCH3 receptor, but with very little accumulation of NOTCH3 NEXT or ICD (Fig. 3A). In contrast, there was less TMIC in NOTCH3^L1519P^-expressing cells in the Notch-off situation, but instead a band corresponding to NOTCH3 NEXT/ICD (Fig. 3A, see also Fig. 1B). This suggests that S2 processing also occurs in NOTCH3^L1519P^ in the absence of ligand activation. In keeping with this notion, blockade of S2 cleavage by treatment of the cells with the ADAM10 secretase inhibitor GI254023X reduced the accumulation of the NEXT/ICD band in the NOTCH3^L1519P^-expressing cells (Fig. 3A). As the NEXT moiety is rapidly processed to the ICD form by the γ-secretase-mediated S3 cleavage (see Fig. 1A), and ICD has a short half-life because of rapid proteasome-mediated degradation (Öberg et al., 2001; Zhang et al., 2017), we explored the effect of treating the cells with the γ-secretase inhibitor N-[N-(3,5-difluorophenacetyl)-L-alanyl]-S-phenylglycine t-butyl ester (DAPT) to block the transition of the NEXT into the ICD form. DAPT treatment resulted in a stronger NEXT/ICD band in NOTCH3^L1519P^-expressing cells (Fig. 3A), indicating that blockage of S3 cleavage leads to accumulation of NEXT, whereas the TMIC level was unaffected in the wild-type NOTCH3-expressing cells. The low levels of TMIC from NOTCH3^L1519P^ may thus be caused by enhanced S2 cleavage in the absence of ligand activation. In support of this notion, cycloheximide chase experiments demonstrated that the TMIC from NOTCH3^L1519P^ in the Notch-off situation was turned over more rapidly than the TMIC from the wild-type NOTCH3 receptor (Fig. 3B). In all, these experiments reveal enhanced and ligand-independent S2 processing of NOTCH3^L1519P^.

### Relocalization of NOTCH3^L1519P^ in the dipartite ECD-TMIC state coupled with exacerbated export of the ECD from the cell

**Fig. 4.**
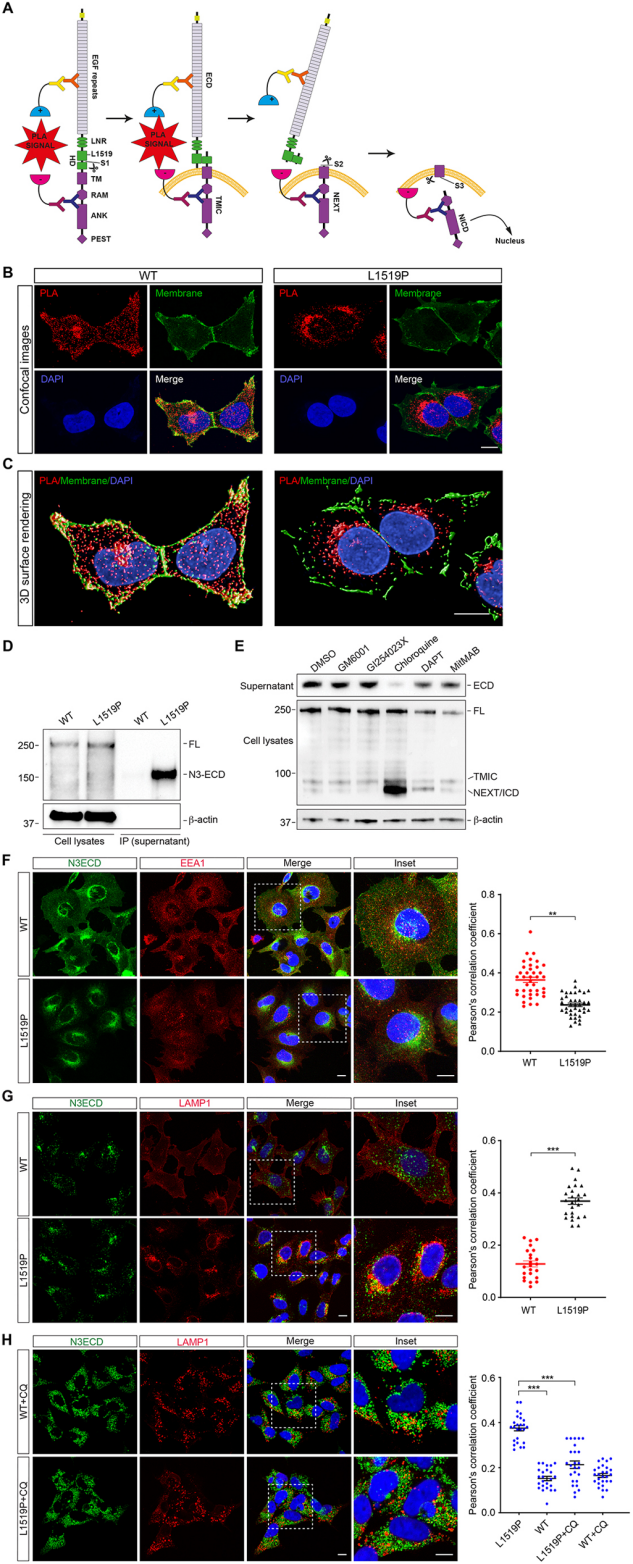
**Relocalization of the ECD-TMIC heterodimer and exacerbated export of NOTCH3^L1519P^ ECD into the cell medium.** (A) Schematic representation of the proximity ligation assay (PLA) identifying the ECD-TMIC heterodimer. (B) PLA (red) was performed on wild-type or NOTCH3^L1519P^-expressing cells, using mouse anti-ECD 1E4 and rabbit anti-NOTCH3 ICD antibodies. The cell membrane (green) was directly labeled with anti-Na/K ATPase-Plasma Membrane Marker (Alexa Fluor^®^ 488). (C) 3D surface rendering of the PLA and cell membrane staining. (D) Immunoprecipitation of supernatants from cells expressing wild-type NOTCH3 or NOTCH3^L1519P^ mutation using V5-agarose beads followed by western blot analysis using anti-V5 antibody. β-actin levels were used as a loading control. (E) Western blot analysis of the conditioned media from cells expressing NOTCH3^L1519P^ treated with dimethyl sulfoxide (DMSO), GM6001, GI254023X, chloroquine, DAPT and MitMAB, as indicated. β-actin levels were used as a loading control for whole-cell extracts. (F) Immunocytochemistry for NOTCH3 ECD (green) and the endosomal marker EEA1 (red). (G) Immunocytochemistry for NOTCH3 ECD (green) and LAMP1 (red). (H) Immunocytochemistry for NOTCH3 ECD (green) and LAMP1 in cells treated with chloroquine (CQ). The plots show the Pearson's correlation coefficiency for the colocalization analysis (*n*=3). Significance was calculated using unpaired Student's *t*-test, **P<10^−2^, ***P<10^−3^. Scale bars: 10 µm.

We next explored the possibility that the enhanced ligand-independent S2 cleavage and lack of NOTCH3^L1519P^ at the cell surface might be due to destabilization of the dipartite state of the receptor, i.e. the ECD-TMIC heterodimer, because the mutation is located in the conserved heterodimerization region (Fig. 1A) and may thus affect stability of the ECD and TMIC interaction. To address this, we established a proximity ligation assay (PLA) that records the proximity of the ECD and TMIC moieties, using antibodies against the ECD and TMIC domains, respectively (Fig. 4A). The ECD-TMIC PLA revealed a more widespread ECD-TMIC interaction for wild-type NOTCH3 compared to NOTCH3^L1519P^, for which much of the PLA signal localized to aggregates in a cytoplasmic area bordering the cell nucleus (Fig. 4B). Three-dimensional (3D) rendering analysis confirmed that the ECD-TMIC wild-type heterodimer was localized at the plasma membrane and in the cytoplasm, whereas the ECD-TMIC mutant heterodimer was predominantly observed in the cytoplasm near the cell nucleus (Fig. 4C). Together, these results indicate that the L1519P mutation leads to relocalization and possible destabilization of the ECD-TMIC heterodimer early during the transport to the cell surface, and indicate the absence of a dipartite NOTCH3^L1519P^ receptor at the cell surface.

The fact that the L1519P mutation shifted the localization of the ECD-TMIC heterodimer, combined with absence of the mutated receptor at the cell surface, led us to explore the fate of the NOTCH3^L1519P^ ECD. To test whether it was exported from the cell, we analyzed cell culture medium from cells expressing NOTCH3^L1519P^ or wild-type NOTCH3. Medium from the NOTCH3^L1519P^-expressing cells not activated by ligand contained considerably more ECD (Chapman et al., 2006) than medium from wild-type NOTCH3 cells, which did not contain measurable amounts of ECD (Fig. 4D).

Although export of NOTCH3^L1519P^ ECD to the cell medium was independent of ligand activation, we next asked whether it was dependent on S2 cleavage, endocytosis or autophagosome-lysosome function. To test this, cells expressing NOTCH3^L1519P^ were treated with GM6001 (a broad-spectrum matrix metalloproteinase inhibitor blocking S2 cleavage), MitMAB (a dynamin I/II inhibitor blocking endocytosis), GI254023X (to block S2 cleavage) and chloroquine (which inhibits fusion of the autophagosome). Treatment with GM6001, GI254023X, DAPT or MitMAB did not affect export of the ECD from NOTCH3^L1519P^ to the cell medium, indicating that S2 cleavage and endocytosis were not required for ECD export (Fig. 4E). In contrast, chloroquine strongly reduced the amount of NOTCH3^L1519P^ ECD in the medium (Fig. 4E). Chloroquine treatment also resulted in an accumulation of a NEXT/ICD band in whole-cell extracts from the NOTCH3^L1519P^-expressing cells (Fig. 4E).

The effect of chloroquine treatment on export of the ECD from NOTCH3^L1519P^ prompted us to analyze the localization of wild-type NOTCH3 and NOTCH3^L1519P^ protein to endosomes and lysosomes. NOTCH3^L1519P^ showed reduced localization to early endosomes, compared to wild-type NOTCH3, using EEA1 as an early endosome marker and analyzed using Pearson's correlation coefficient (Fig. 4F). In contrast, when colocalization with lysosomes was analyzed using LAMP1 as a lysosomal marker, NOTCH3^L1519P^ colocalized more extensively with LAMP1 compared to wild-type NOTCH3 (Fig. 4G). Treatment with chloroquine decreased colocalization between NOTCH3^L1519P^ and lysosomal LAMP1 (Fig. 4H). In sum, these data indicate that the heterodimeric ECD-TMIC from NOTCH3^L1519P^ is relocalized in the cell, which may lead to the exacerbated export of its ECD from the cell in an autophagosome-lysosome fusion-dependent mode.

### NOTCH3^L1519P^ produces NOTCH3 ICD in a ligand-independent manner, leading to enhanced Notch downstream signaling output

**Fig. 5.**
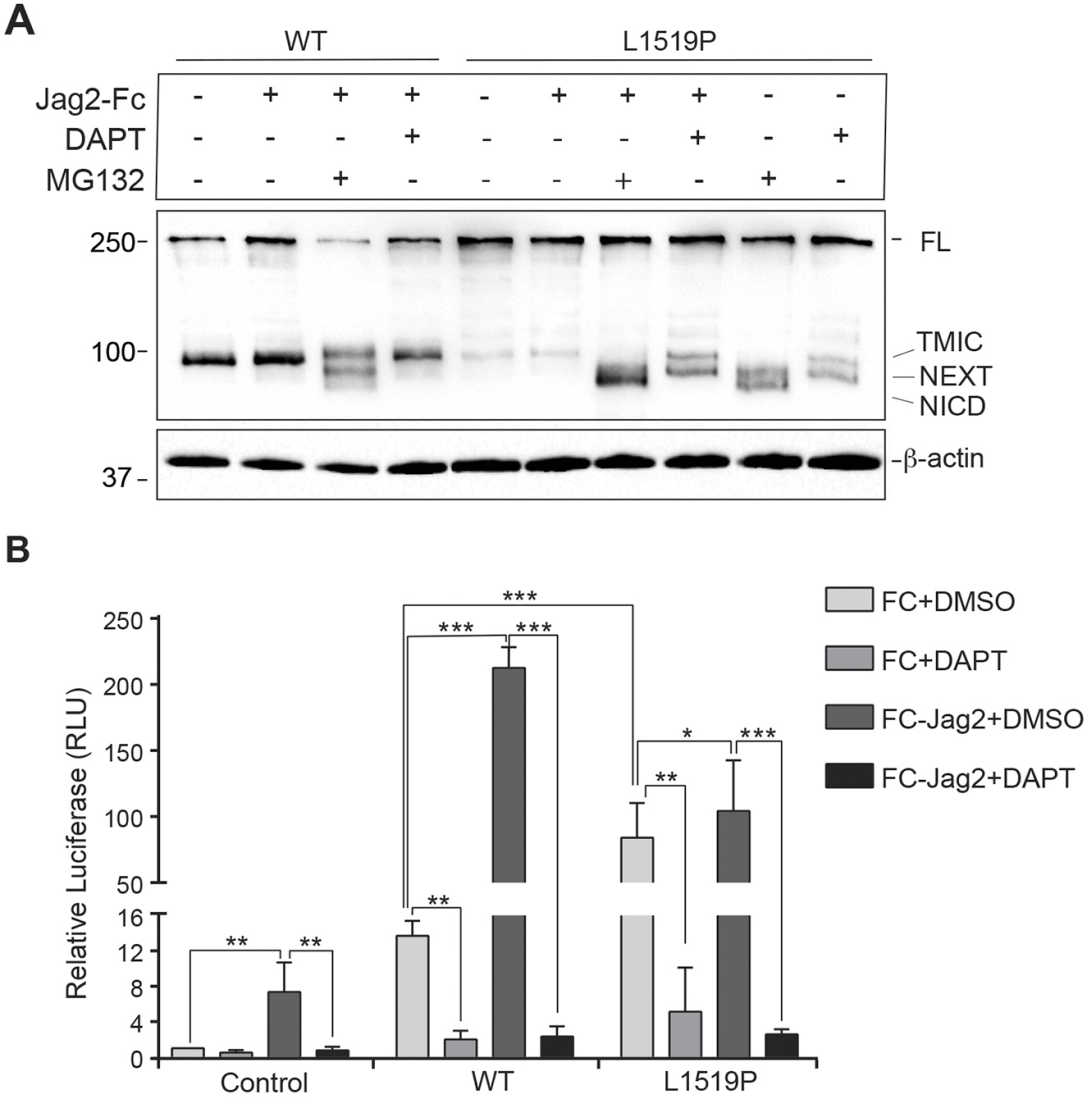
**NOTCH3^L1519P^ produces ICD in a ligand-independent manner.** (A) Western blot analysis of FL, TMIC and NEXT and ICD fragments from cell extracts using an anti-NOTCH3 ICD antibody, following activation of the cells by immobilized jagged2 (Jag2-Fc) and treatment with the γ-secretase inhibitor DAPT or MG132, as indicated. (B) NIH3T3 cells were transfected with wild-type, NOTCH3^L1519P^ or control plasmid together with 12XCSL-luc reporter and β-gal plasmids and cultured on immobilized jagged2 (Jag2) in combination with treatment by DMSO or DAPT, as indicated (*n*=3). Statistical analysis was performed from three experiments using paired Student's *t*-test, *P<0.1, **P<10^−2^, ***P<10^−3^, RLU, relative luminescence units.

To learn how ligand-mediated activation affected NOTCH3^L1519P^ processing and downstream signaling, we first subjected cells expressing wild-type NOTCH3 or NOTCH3^L1519P^ receptor to ligand activation by culturing the cells on immobilized jagged2 ligand. As expected, ligand activation of the wild-type NOTCH3 receptor led to the production of NEXT and a small amount of ICD when MG132 was supplemented to block proteasome-mediated degradation of NOTCH3 ICD (Öberg et al., 2001) (Fig. 5A), indicating that NEXT and ICD were rapidly turned over when proteasome activity was not inhibited. Blockade of S3 processing using DAPT under ligand-activating conditions abolished the ICD band while sustaining a small amount of NEXT (Fig. 5A), indicating that NEXT is rapidly degraded by the proteasome. For cells expressing NOTCH3^L1519P^, there was less TMIC (Fig. 5A), in keeping with the results presented in Fig. 1B. Treatment with MG132 resulted in a strong accumulation of NEXT, and in particular of ICD, and this accumulation was interestingly observed both in the ‘Notch-on’ and Notch-off situations, i.e. both with and without ligand stimulation (Fig. 5A). Treatment with DAPT resulted in an accumulation of NEXT, also both in the Notch-on and Notch-off states. Collectively, these data suggest an enhanced S2 cleavage of NOTCH3^L1519P^, leading to production of a NEXT moiety, which accumulates when S3 cleavage is blocked, but which otherwise rapidly turns over into the ICD form in a ligand-independent manner.

To learn whether the enhanced S2 processing and emergence of a NOTCH3 ICD even under non-ligand activation conditions resulted in enhanced Notch downstream signaling, NIH3T3 cells were transfected with a Notch reporter system, 12X-CSL-luc, to record Notch activation immediately downstream of the Notch receptor (Chapman et al., 2006). In control NIH3T3 cells not transfected with wild-type NOTCH3 or NOTCH3^L1519P^, there was no induction of 12X-CSL-luc activity irrespective of ligand activation or not, while cells expressing wild-type NOTCH3 receptor showed low activity in the Notch-off state and robust reporter activation upon ligand activation, which was abrogated by DAPT treatment (Fig. 5B). In the NOTCH3^L1519P^ receptor-expressing cells, in contrast, the Notch reporter was activated also in the Notch-off state, almost to the same extent as under ligand-activating conditions, and DAPT in both cases abrogated the activation (Fig. 5B). These data show that the NOTCH3^L1519P^ constitutively activates Notch downstream signaling.

### NOTCH3^L1519P^ upregulates PDGFRB expression

**Fig. 6.**
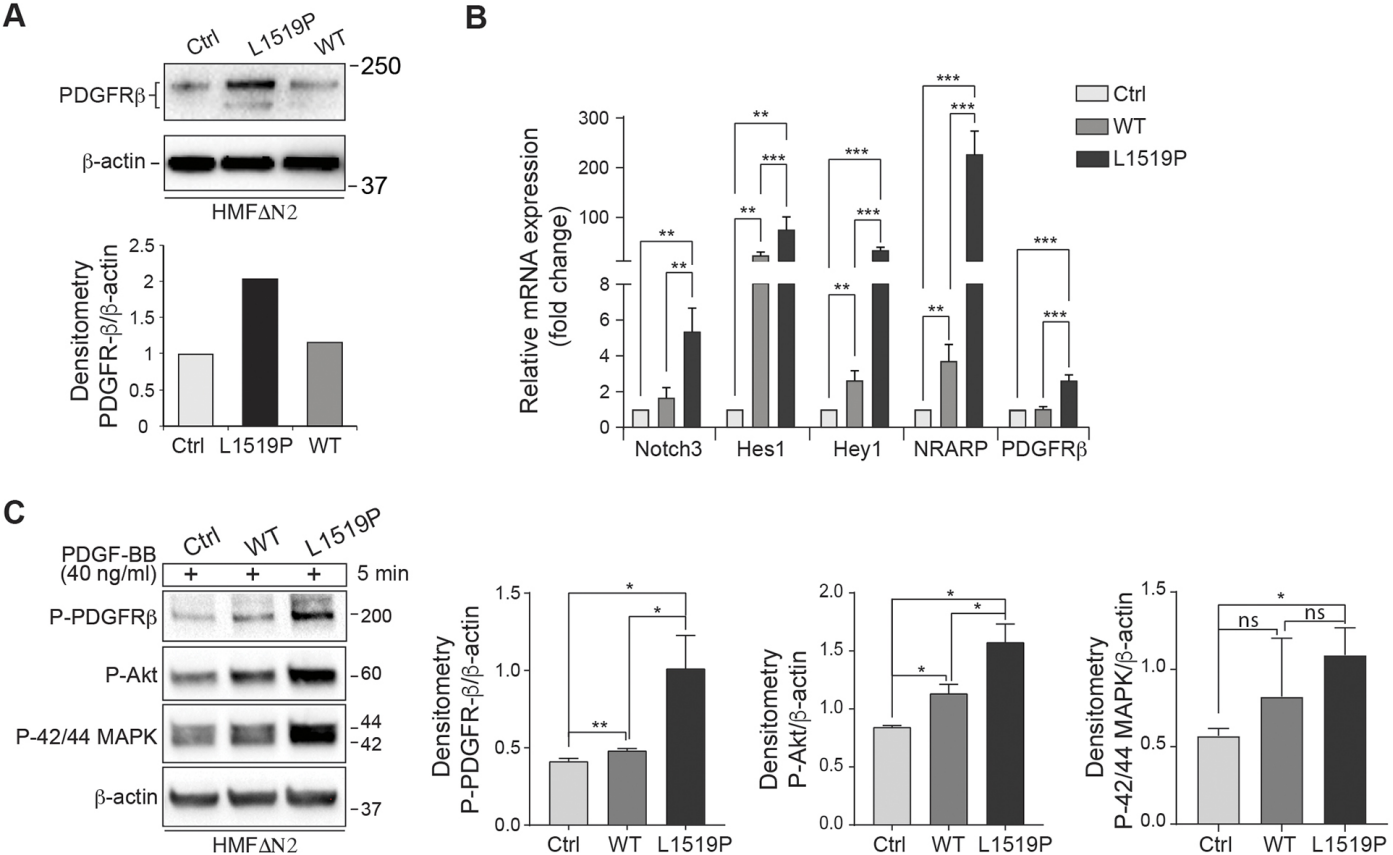
**NOTCH3^L1519P^ hyperactivates expression of Notch downstream genes.** (A) Western blot analysis of PDGFRB expression in HMFΔN2 cells expressing wild-type NOTCH3 (WT) or NOTCH3^L1519P^ (L1519P) compared to the parental HMFΔN2 cells (Ctrl). The graph shows PDGFRB expression normalized to β-actin using ImageJ. (B) Quantitative real-time PCR analysis of the Notch downstream target genes *NOTCH3*, *HES1*, *HEY1*, *NRARP* and *PDGFRB* from wild-type or NOTCH3^L1519P^-expressing HMFΔN2 cells. (C) Western blot analysis of PDGFRB, AKT and p42/44 MAPK phosphorylation upon PDGF-BB stimulation as indicated. The blot with p42/44 MAPK was stripped and re-probed with the β-actin antibody for a loading control. Relative levels were normalized to β-actin using ImageJ (*n*=3). Statistical significant was analyzed from three experiments using paired Student's *t*-test, *P<0.1, **P<0.01, ***P<0.001; ns, not significant.

As dysregulated PDGF signaling has been linked to IMF (Arts et al., 2017; Cheung et al., 2013; Martignetti et al., 2013; Mudry et al., 2017), we next sought to explore a possible link between Notch and PDGF signaling in fibroblasts, the presumed cell type of origin for IMF. To this end, we conducted the experiments in a fibroblast cell line derived from human mammary fibroblast (HMF) tumor stromal fibroblasts, in which we had previously removed the *NOTCH2* gene by CRISPR/Cas9 cells to generate a fibroblast cell line with very low endogenous Notch signaling due to the removal of NOTCH2 (HMFΔN2) (Strell et al., 2019), but with the endogenous *NOTCH3* gene retained, thus more closely mimicking the human IMF heterozygous situation. Wild-type or mutant *NOTCH3^L1519P^* genes were then stably introduced into the HMFΔN2 cells via insertion into the AAVS1 locus, as described above. Notch signaling has previously been reported to activate expression of PDGFRB in vascular smooth muscle cells (Jin et al., 2008), and we therefore analyzed how levels of PDGFRB were regulated by wild-type NOTCH3 and NOTCH3^L1519P^ expression. HMFΔN2 cells expressed a low level of PDGFRB protein, and a similar level was observed in HMFΔN2 cells expressing wild-type NOTCH3, whereas the PDGFRB level was higher in NOTCH3^L1519P^-expressing HMFΔN2 cells (Fig. 6A). To assess whether the observed upregulation was a result of transcriptional activation, we assessed the mRNA levels of *PDGFRB*, as well as those of four well-established Notch downstream genes (*HES1*, *HEY1*, *NOTCH3* and *NRARP*). *HES1*, *HEY1*, *NOTCH3* and *NRARP* expression was upregulated by wild-type NOTCH3 expression and further elevated in cells expressing NOTCH3^L1519P^ (Fig. 6B). For *PDGFRB*, there was no upregulation of expression in the wild-type NOTCH3-expressing cells, whereas there was increased expression in the NOTCH3^L1519P^-expressing cells (Fig. 6B), in line with the protein data in Fig. 6A.

To assess the possible effects of NOTCH3^L1519P^ on PDGFRB expression and also further downstream in the PDGFRB signaling cascade, we monitored the levels of PDGFRB autophosphorylation and phosphorylation of the downstream effectors AKT and MAPK (p42 and p44 MAPK). Upon treatment of cells expressing wild-type NOTCH3 or NOTCH3^L1519P^ with PDGF-BB ligand, we observed an increased level of PDGFRB autophosphorylation in the NOTCH3^L1519P^-expressing cells as well as increased AKT phosphorylation (Fig. 6C). p42/44 MAPK phosphorylation was likewise augmented in the NOTCH3^L1519P^-expressing cells compared to control cells, but this was not the case for the NOTCH3^L1519P^-expressing cells (Fig. 6C). Collectively, these results suggest that NOTCH3^L1519P^ elicits elevated PDGFRB downstream signaling, and, in line with this notion, there was also an increase in cell proliferation [as judged by increased Ki67 (MKI67) expression] in the PDGF-BB-stimulated NOTCH3^L1519P^-expressing cells but not the wild-type NOTCH3-expressing cells (Fig. S3).

**Fig. 7.**
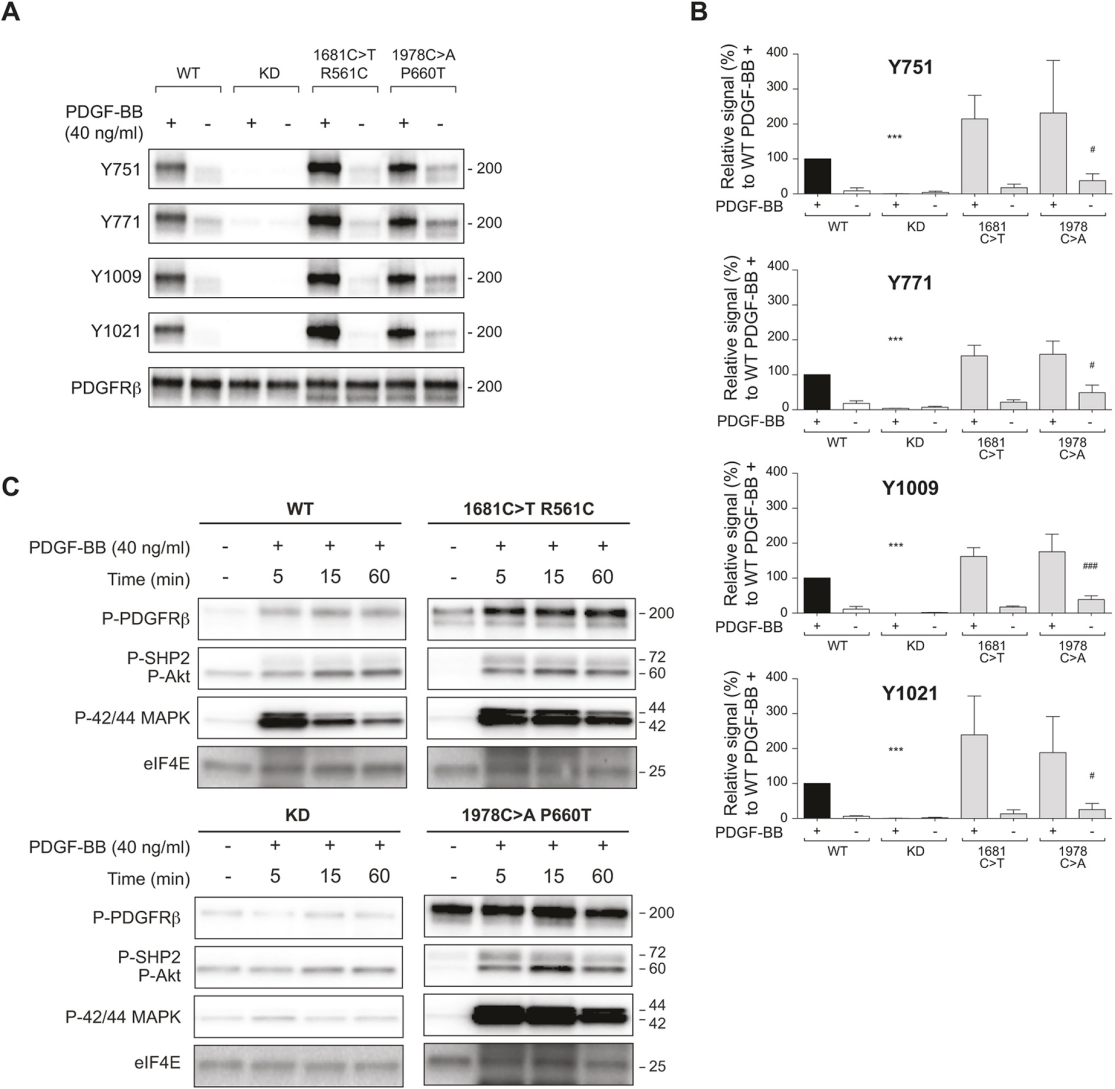
**PDGFRB IMF mutations are gain-of-function mutations.** (A) Analysis of phosphorylation levels from different PDGRFB mutants after stimulation with PDGF-BB, as indicated. (B) Quantification of phosphorylation of specific tyrosine residues (Y751, Y771, Y1009, Y1021) in the various PDGFRB mutants, as indicated (*n*=3). (C) Analysis of phosphorylation levels of PDGFRB, SHP2, Akt, p42/44 MAPK and eIF4E (as control) at different time points after PDGF-BB stimulation (0, 5, 15 and 60 min), as indicated. ***P<0.001 compared to the stimulated WT control (black bars); ^#^*P*<0.05 and ^###^*P*<0.001 compared to unstimulated WT control (unpaired two-tailed Student's *t*-test). KD, knockdown.

If upregulation of PDGFRB is a critical part of the pathogenic function of NOTCH3^L1519P^, it may be assumed that the dominant PDGFRB mutations associated with IMF are gain-of-function mutations. To test this, we assessed the nature of the PDGFRB R561C and P660T IMF mutations. Transfection of PDGFRB^R561C^ and PDGFRB^P660T^ into HEK 293T cells revealed that they, upon PDGF-BB ligand activation, exhibited a higher level of receptor phosphorylation compared to control PDGFRB, whereas a kinase-dead version of PDGFRB (carrying the L658P mutation) did not elicit receptor phosphorylation (Fig. 7A). PDGFRB dimerizes and becomes autophosphorylated upon ligand binding, and autophosphorylation can occur on as many as 13 cytoplasmic tyrosine residues (Andrae et al., 2008; Tallquist and Kazlauskas, 2004). We next analyzed the phosphorylation of tyrosine residues 751, 771, 1009 and 1021, and, in all cases, phosphorylation was enhanced in the PDGFRB^R561C^ and PDGFRB^P660T^ mutants compared to wild-type PDGFRB (Fig. 7B). Finally, activation of downstream signaling events was analyzed for the various PDGFRB mutants. Increased phosphorylation of 42/44 MAPK and AKT was observed in cells transfected with the PDGFRB^R561C^ and PDGFRB^P660T^ mutants (Fig. 7C). These data are in agreement with a previous report indicating that the PDGFRB mutations found in IMF patients are gain-of-function mutations (Mudry et al., 2017). Together, these data indicate that NOTCH3^L1519P^ upregulates expression of PDGFRB and that PDGFRB IMF mutations are gain-of-function mutations.

## DISCUSSION

IMF has been linked to mutations in *PDGFRB* (Arts et al., 2017; Cheung et al., 2013; Martignetti et al., 2013; Mudry et al., 2017), but patients with mutations in the NOTCH3 receptor gene (*NOTCH3^L1519P^*) have also been identified (Arts et al., 2017; Martignetti et al., 2013). In this study, we decode the molecular consequences of the *NOTCH3^L1519P^* mutation and demonstrate that it produces a ligand-independent hyperactivated form of the NOTCH3 receptor. The hyperactive nature of NOTCH3^L1519P^ is supported by an elevated downstream signaling output, both with regard to Notch reporter activity and expression of the Notch downstream genes *HES1*, *HEY1*, *NOTCH3* and *NRARP*. In line with an increased signaling output, we also observed enhanced production of the ICD from the NOTCH3^L1519P^ protein. Interestingly, hyperactive signaling was shown to occur in a ligand-independent manner, as evidenced by the NOTCH3^L1519P^ receptor generating the NEXT and ICD forms also in the Notch-off state, i.e. without ligand stimulation, and the mutated receptor never being observed at the cell surface, thus precluding it from normal ligand-dependent activation.

How can the lack of cell surface presence combined with hyperactive signaling be reconciled? Although we do not have a complete understanding of this, the data support a scenario in which both the proteolytic processing and the intracellular routing of the NOTCH3^L1519P^ receptor are altered. Notch receptors undergo a series of proteolytic processing steps (Fig. 1A), and there are several lines of evidence pointing to enhanced and ligand-independent S2 processing of NOTCH3^L1519P^. First, there is less of the S1-processed form, TMIC, of the NOTCH3^L1519P^ protein, and the mutated TMIC has a shorter half-life, indicating that it may be more rapidly processed. Second, the amount of the S2-cleaved form, NEXT, is increased, and NEXT is also generated in the absence of ligand, suggesting ligand-independent S2 processing. This spontaneous ligand-independent cleavage is likely still conducted by ADAM proteins, as the generation of NEXT was blocked by the ADAM inhibitor GI254023X. Third, in line with the spontaneous production of NEXT, NOTCH3 ICD, which is constitutively generated from NEXT by the γ-secretase complex, was also produced under Notch-off conditions when proteasome-mediated degradation was blocked by MG132. The hypothesis that S2 processing is affected is corroborated by the notion that the L1519P mutation is located in the heterodimerization domain (Fig. 1A) and may thus lower the affinity between the ECD and TMIC moieties, facilitating S2 cleavage. In line with such an effect, the L1519P mutation is likely to destabilize the receptor (−1.8 kcal/mol according to the DUET software) (Pires et al., 2014). Collectively, these observations demonstrate that NOTCH3^L1519P^ is constantly ‘on’, generating NEXT and ICD. The observed increase in ligand-independent signaling from NOTCH3^L1519P^ is also in keeping with the results of a previous study (Xu et al., 2015).

Disturbed intracellular routing of NOTCH3^L1519P^ is manifested by increased retention of NOTCH3^L1519P^ in the ER as well as in the lysosome, combined with reduced amounts in early endosomes. The PLA reporting on NOTCH3 receptors in an uncleaved or dipartite, heterodimeric state, with the ECD and TMIC in close proximity, also revealed an altered intracellular distribution for NOTCH3^L1519P^, with aggregates localized close to the cell nucleus. Together, these findings may argue for an initial problem in ER-to-Golgi routing, which results in aberrant shunting of the mutated receptors from the ER to the lysosomes. There is indeed emerging evidence for transport systems directly between ER/Golgi and lysosomes, where cells under certain conditions promote the formation of ER-lysosome contacts, facilitating the transfer of ER-associated proteins to the lysosomal surface (Lie and Nixon, 2019). It is also increasingly realized that lysosomes, in addition to their role in protein degradation and autophagy, can act as a signaling hub and thus have both degrading and signaling functions (Lie and Nixon, 2019). It may thus be hypothesized that the aberrant and ligand-independent S2 processing occurs in the lysosome, a notion supported by the exacerbated export of the mutated NOTCH3 ECD from the cell being abrogated by chloroquine, which blocks autophagosome-lysosome fusion. Aberrant S2 processing may also be facilitated by autocatalytic cleavage, which should be favored by the low pH in the lysosome (Fairchild et al., 2001; Lidell et al., 2003; Thuveson and Fries, 2000). It is, however, of note that receptor processing occurs via the S2 and S3 sites, as it was sensitive to ADAM and γ-secretase inhibitors. In a broader *NOTCH3* mutation and disease context, it is interesting to observe that a similar leucine-to-proline mutation in amino acid 1515, i.e. located only four amino acid residues away from the IMF mutation at position 1519, produces a distinct disease outcome. A patient with the *NOTCH3^L1515P^* mutation, which also generates a receptor with enhanced signaling and secretion of ECD, instead developed cerebral small vessel disease (Fouillade et al., 2008). The reason why two so closely related NOTCH3 mutations give different disease outcomes is not understood, and it will be interesting to learn whether IMF patients carrying the NOTCH3^L1519P^ mutation develop vascular problems later in life and, conversely, whether IMF would be more frequently occurring in patients with NOTCH3 mutations linked to vascular disease.

As IMF mutations are found in both the *NOTCH3* and *PDGFRB* genes, it is of interest to learn whether Notch and PDGF signaling are linked. Our data show that *in vitro* expression of NOTCH3^L1519P^, but not wild-type NOTCH3, causes an upregulation of PDGFRB expression, both at the mRNA and protein levels, in fibroblasts, i.e. the cell type that is the likely origin for IMF tumors. This reveals that Notch acts epistatically over PDGFRB expression, corroborating a previously observed Notch–PDGFRB axis in vascular smooth muscle cells (Jin et al., 2008). Together with our confirmation that the IMF mutations PDGFRB^R561C^ and PDGFRB^P660T^ are gain-of-function mutations (Cheung et al., 2013; Martignetti et al., 2013), the data argue for a Notch–PDGF axis in IMF, in which a hyperactive NOTCH3 mutation causes elevated PDGFRB expression, or, alternatively, elevated PDGF signaling is achieved by gain-of-function mutations in PDGFRB. In line with this hypothesis, it has previously been observed that elevated levels of PDGFRB have been noted in pediatric fibromatosis and myofibromatosis (Gibson et al., 2007).

The ligand-independent hyperactive signaling from the NOTCH3^L1519P^ receptor has implications for Notch therapy considerations for IMF (Andersson and Lendahl, 2014). One consequence of the ligand-independent signaling is that Notch activation may occur in places and at time points at which a wild-type ligand-dependent NOTCH3 receptor would not normally be activated, including cell contexts in which no Notch ligands are expressed. Second, the notion that NOTCH3 is itself a target gene of the augmented activation by NOTCH3^L1519P^ suggests that the aberrant activation via NOTCH3^L1519P^ could result in a loop, whereby an initial expression of the mutant receptor leads to further elevated receptor levels. Finally, the observation that NOTCH3^L1519P^ acts ligand independently and never reaches the cell surface suggests that antibody-based therapies that block NOTCH3 function via clamping the NRR (Wu et al., 2010) or affect ligand-receptor interaction (Chung et al., 2017; Lafkas et al., 2015; Tran et al., 2013) may not be effective on this type of Notch mutation. Instead, approaches based on small molecules interfering with Notch function further down in the signaling cascade (Andersson and Lendahl, 2014) may be more appropriate candidates for future therapy for IMF patients with *NOTCH3^L1519P^* mutations.

In conclusion, our study identifies a NOTCH3–PDGFRB axis in IMF, in which Notch signaling is epistatic over PDGFRB and activating mutations can occur in both the *NOTCH3* and *PDGFRB* genes. This information is of relevance for therapy considerations for IMF and for understanding how Notch signaling can get derailed by missense mutations in a Notch receptor.

## MATERIALS AND METHODS

### Establishment of CRISPR knockout and knock-in cell lines

To generate the HEK 293T cell line in which *NOTCH1*, *NOTCH2* and *NOTCH3* were ablated (HEK 293T ΔN1-3), single-guide RNA (sgRNA) targeting *NOTCH1*, *NOTCH2* and *NOTCH3* (Table S1) was cloned into the guide RNA (gRNA) Cas9 vector (Addgene #62988). HEK 293T cells were transfected with the gRNA vector, and puromycin dihydrochloride (Sigma-Aldrich) at 1 µg/ml was used for selection. Single-cell colonies were isolated and subjected to western blotting as previously described (Strell et al., 2019). To integrate the V5-Notch3 and V5-NOTCH3^L1519P^ sequence into the AAVS1 locus in HEK 293T ΔN1-3 cells, the AAVS1-T2 gRNA vector (Addgene #41818) was co-transfected with the AAVS1.V5-Notch3 or AAVS1.V5-NOTCH3^L1519P^ donor vector. The transfected cells were cultured for 5 days; thereafter, 1 µg/ml puromycin was used to screen single-cell clones.

### DNA constructs

The detailed description of the cloning work is provided in the Supplementary Materials and Methods.

### Cell culture and treatments

HEK 293T, HEK 293T ΔN1-3 and its derived NOTCH3- and PDGFRB-expressing cell lines, NIH3T3 and HMFΔN2 cells were maintained in Dulbecco's modified Eagle medium (DMEM), supplemented with 10% fetal bovine serum and 1% penicillin-streptomycin (Life Technologies) at 37°C in a humidified 5% CO_2_ atmosphere. All transfections were conducted using Lipofectamine^®^ 2000 or Lipofectamine^®^ Plus (Life Technologies) according to the manufacturer's instructions. Detailed description of PDGF-BB treatment is provided in the Supplementary Materials and Methods. All reagents used are listed in Table S2.

### Image streaming

Cells were immunostained with an antibody against the NOTCH3 ECD (1E4) and sorted by an image streamer, in which cells are simultaneously imaged and sorted (image flow cytometry). The detailed description is provided in the Supplementary Materials and Methods and antibodies are listed in Table S3.

### Luciferase assay

NIH3T3 cells were transfected with 12×CSL-luc and CMV-β-galactosidase together with wild-type NOTCH3, L1519P plasmid or pcDNA3 vector as a control. For more detail, see the Supplementary Materials and Methods.

### PLA

PLA was performed using the Duolink *In Situ* Detection Reagents Red Kit (DUO92008, Sigma-Aldrich), according to the manufacturer's protocol. Briefly, HEK 293T ΔN1-3 V5-NOTCH3 and HEK 293T ΔN1-3 V5-NOTCH3^L1519P^ cells were cultured for 24 h on coverslips and fixed with 4% paraformaldehyde in PBS for 10 min, followed by blocking with 5% bovine serum albumin (BSA) in 0.1% Triton X-100 for 1 h at 37°C. Primary antibodies, anti-1E4 (mouse, Sigma-Aldrich) and anti-NOTCH3 (rabbit, Abcam), were incubated overnight at 4°C (see Table S3 for antibody dilutions). After washes with PBS, the slides were incubated with the PLA probes anti-mouse MINUS (DUO92004, Sigma-Aldrich) and anti-rabbit PLUS (DUO92002, Sigma-Aldrich), according to the manufacturer's protocol. The plasma membrane was stained using the anti-Na/K ATPase-Plasma Membrane Marker (Alexa Fluor^®^ 488) (Abcam). Subsequently, the coverslips were washed with PBS and mounted with Duolink *in situ* mounting medium containing 4′,6-diamidino-2-phenylindole (DAPI).

### Activation of Notch signaling by immobilized ligand

Cell culture plates were coated with recombinant human jagged2 Fc chimeric protein (#P78504, R&D Systems) at 0.5 µg/ml, with IgG Fc fragment as control. Duration of treatments is described in the Supplementary Materials and Methods.

### Western blot analysis

The detailed description is provided in the Supplementary Materials and Methods and antibodies are listed in Table S3.

### Immunoprecipitation

Cells were incubated in DMEM containing 20 ng/ml doxycycline for 2 days. Conditioned medium was collected and cleared by centrifugation at 3000 ***g*** for 5 min and incubated with anti-V5 agarose (Novus Biologicals) overnight, followed by washing of the beads with lysis buffer containing 10 mM Tris-HCl (pH 7.4), 150 mM NaCl, 0.5% Nonidet P-40 and protease inhibitor (Roche). Bound proteins were detected by immunoblotting using anti-V5 antibody (Life Technologies).

### RNA extraction and real-time PCR

RNA extraction and cDNA synthesis were accomplished as previously described (Chapman et al., 2006). Real-time PCR analysis was carried out on a 7500 Fast Real-Time PCR system with Fast SYBR Green Master Mix (Applied Biosystems) according to the manufacturer's protocol. Primers for qPCR are listed in Table S1.

### Cell proliferation assay

The detailed description is provided in the Supplementary Materials and Methods and antibodies are listed in Table S3.

### Immunocytochemistry and confocal microscopy

Cells were plated on glass coverslips for 24 h, then fixed for 10 min at room temperature with 4% paraformaldehyde. Cells were permeabilized with 0.1% Triton X-100 in PBS followed by blocking with 0.1% Triton X-100 and 2% BSA in PBS for 1 h. Primary antibodies were incubated at 4°C overnight, and, after washes in PBS, samples were incubated with secondary antibodies for 1 h at room temperature and mounted with Vectashield medium with DAPI (Vector Laboratories). Images were captured by confocal microscopy (LSM 880 META, Carl Zeiss AG). Image analysis was carried out using ImageJ (National Institutes of Health) and Adobe Photoshop software. Antibodies are listed in Table S3.

### Statistical analysis

GraphPad Prism 8.4.3 was used to generate the bar graphs, and results were analyzed by Student's *t*-test to assess the statistical differences between experimental groups. *P*≤0.05 was considered statistically significant. Error bars represent s.d. Pearson's correlation coefficient was carried out as previously described (Adler and Parmryd, 2010).

## Supplementary Material

Supplementary information
