# Supplementary material for: The infantile myofibromatosis NOTCH3 L1519P mutation leads to hyperactivated ligand-independent Notch signaling and increased PDGFRB expression
Source: Dis Model Mech. 2021 Feb 24;14(2):dmm046300. doi: 10.1242/dmm.046300 (PMC7927659; doi:10.1242/dmm.046300)
Supplement: Supplementary information [file dmm-14-046300-s1.pdf]

Supplementary information

Materials and Methods

DNA constructs

The NOTCH3^{L1519P} mutation (c.4556 C>T) was generated by using the QuikChange XL Site-Directed Mutagenesis Kit (Agilent) with the primer pairs in Supplementary Table 1, according to the manufacturer's instructions. The resulting mutation was verified by DNA sequencing. The V5-Notch3 and V5-NOTCH3^{L1519P} plasmids were generated by using oligonucleotides encoding the V5 epitope sequences and fused *in frame* in the coding region of wild type NOTCH3 gene or NOTCH3^{L1519P} mutation at the N-terminus by PCR using the KOD Xtreme™ Hot Start DNA Polymerase (Merck) To introduce the AAVS1 sites in the Notch3 knock-in expression vector, the sequences of V5-Notch3 and V5-NOTCH3^{L1519P} were amplified by PCR using the Taq polymerase as described above and cloned into the HincII site in the AAVS1-TRE3G-EGFP donor vector (Addgene, #52343) to obtain AAVS1.V5-Notch3 and AAVS1.V5-NOTCH3^{L1519P}.

Constructs of human wild-type (WT) and kinase-dead (KD) PDGFRβ subcloned into the pcDNA 3 vector (Life Technologies) were kindly provided by C.H. Heldin (Ludwig Institute for Cancer Research, Uppsala, Sweden). The KD construct carries the K634A mutation in which the nucleotide-binding lysine of the protein-tyrosine domain has been changed to alanine [1]. To generate the PDGFR mutants, R561C and P660T, a QuikChange II Site-Directed Mutagenesis Kit was used according to the manufacturer's instructions, with the primers in Supplementary Table 1. The sequence of the full insert was determined for each construct.

Image streaming

Cells were dissociated with TrypIE and blocked with 2% FBS in PBS on ice for 15 minutes, followed by filtering through a 70-µm cell strainer (BD Biosciences) to get single-cell suspensions. Cells were incubated with primary antibody 1E4 for 30 min on ice and after washing with blocking buffer, the cell suspensions were stained with goat anti-mouse Alexa 488 secondary antibody for 30 minutes at r.t in the dark. Cell nuclei were counterstained with DRAQ5 (*red)* before the acquisition. Samples were analyzed using a BD LSR Fortessa X-20 cytometer.

PDGF-BB treatment

Recombinant human PDGF-BB was purchased from R&D Systems. For PDGFRβ autophosphorylation assays, cells were starved in 0% FBS overnight and cooled on ice for 15 min before addition of 40 ng/ml PDGF-BB, conditioned medium or DMEM-vehicle. Cells were treated on ice for 1 hour, then harvested. For time-course analysis, PDGF-BB was directly added to the normal growth medium at 37°C to reach a concentration of 40 ng/ml. Cells were rinsed in ice-cold PBS and snap-frozen at different time-points.

Luciferase assay

NIH3T3 cells were transfected with 12×CSL-luc and CMV-β-galactosidase together with Notch3 wildtype, L1519P plasmid or pcDNA3 vector as a control. DAPT was added at 6 hour post transfection, as indicated. After incubation for 24 hours, the cells were lysed in Cell culture lysis reagent (Promega) and luciferase activity was measured in triplicate by the GloMax® Multi Detection System apparatus (Promega) using the Dual-Glo Luciferase Assay System (Promega) according to the manufacturer's protocol. In all assays, relative luciferase activity was calculated as the ratio of Luciferase values normalized to β-gal levels.

Western blot analysis

Cells were lysed in RIPA buffer supplemented with protease inhibitor cocktail (Complete, Roche). Protein concentrations were determined using the BCA method (Thermo Scientific, Pierce). The lysates were separated in 4–12% Mini-PROTEAN TGX gels (Bio-Rad) or 10% SDS-PAGE, and transferred to nitrocellulose membranes or PVDF membrane using Trans-Blot Turbo transfer system (Bio-Rad). The membranes were after blocking with 5% nonfat milk in PBS, incubated with primary antibody overnight at +4 °C followed by probed with HRP-linked secondary antibody (GE Healthcare). Antibodies are listed in the Supplementary Table 3. When needed, membranes were stripped once using the Antibody stripping buffer (Interchim) and reprobed with different antibodies. Complete removal of the primary and secondary antibodies were checked by incubation of the stripped membrane with the HRP-coupled secondary antibody followed by chemiluminescent substrate (Clarity Western ECL substrate, BioRad or SuperSignal kit, Pierce). A high-resolution CCD camera (ChemiDoc MP, BioRad) was used for signal detection and optimal exposure was determined by the software. For the statistical analysis of band intensity, results from three experiments were included.

Cell proliferation assay

Cells were cultured on glass coverslips in 12-well plates to 30% confluency using the medium described in "Cell culture and treatments", followed by 18 hours in serum-reduced medium containing 5% FBS with 50ng/ml of PDGF-BB. After three washes with PBS, cells were fixed

with 4% PFA and permeabilized with 0,2% Triton X-100 in PBS. Samples were stained with primary rabbit anti-Ki-67 antibody over night at 4°C, followed by incubation with secondary antibody for 1h and counterstained with DAPI. Images were taken at 40x magnification. The numbers of Ki-67positive cells were normalized to DAPI staining.

References:

1 Westermark B, Siegbahn A, Heldin C, Claesson-welsh L. B-type receptor for platelet-derived growth factor mediates a chemotactic response by means of ligand-induced activation of the receptor protein-tyrosine kinase. *Proc Natl Acad Sci U S A* 1990; **87**: 128–132.

Table S1

Primers used for mutagenesis, CRISPR knock-out cell line and qPCR

	Mutagenesis primers	
NOTCH3-L1519P mutation	CTCCTCTGGCGGCGGCAGCACTGTGAG	CTCACAGTGCTGCCGCCGCCAGAGGAG
PDGFRβ-R561C mutation	TTTGGCAGAAGAAGCCATGTTACGAGATCCGATGG	CCATCGGATCTCGTAACATGGCTTCTTCTGCCAAA
PDGFRβ-P660P mutation	TGAGTCACCTTGGGACCCACCTGAACGTG	CACGTTCAGGTGGGTCCCAAGGTGACTCA
	CRISPR knock-out sgRNA targeting sequence	
NOTCH1	GGTGAGACCTGCCTGAATGG	
NOTCH2	GGTGGAGCCTGGAGTACAGG	
NOTCH3	GGCTTCCGTCCAGGCAAGGG	
	qPCR primers	
NOTCH3	AGTTACCCCCAAGAGGCAAGT	TATCTCGGTCACGCTGCAA
β-actin	CCAGAGGCGTACAGGGATAG	CCAACCGCGAGAAGATGA
NRARP	TTCTCCCTCTCCCTCAAATCC	AACTGCAAAACAAGCCGGTT
HES1	TCGTTCATGCACTCGCTGA	AGGCGGACATTCTGGAAATG
HEY1	AAAAAGCCGAGATCCTGCAGA	CCGAAATCCCAAACTCCGATA
PDGFRβ	TTGCCAGTTCCACCTTGAATG	AGTTGTGCCTCAGGCTCTGCTT

Disease Models & Mechanisms • Supplementary information

Table S2

List of reagents

Name	Concentration	Incubation time	Provider
Mg132	10um	overnight	Merck Millipore
γ-secretase inhibitor DAPT	10um	overnight	Merck Millipore
Chloroquine	25um	overnight	Sigma-Aldrich
GM6001	100um	overnight	Sigma-Aldrich
GI254023X	140um	overnight	Sigma-Aldrich
MitMab	1um	overnight	Abcam
Doxycycline	20ng	overnight	Sigma-Aldrich
Cycloheximide	5ug/ml	overnight	Sigma-Aldrich
Fc fragment	1ug/ml	overnight	Sigma-Aldrich
Jagged 2 Fc	1ug/ml	overnight	R&D system
Protein G	50ug/ml	2h	Thermo Fisher Scientific
PDGF-BB	40ng/ml	0, 5, 15, 60 min	R&D Systems

Table S3

List of antibodies

Name of antibody	Dilution	Provider
anti-Notch1 (D1E11)	1:1000	Cell Signaling Technology
anti-Notch2 (D67C8)	1:1000	Cell Signaling Technology
anti-Notch3 (D11B8),	1:1000	Cell Signaling Technology
anti-PDGFRB (C82A3)	1:1000	Cell Signaling Technology
anti-NOTCH3/N3ECD(1E4)	1:1000	Sigma-Aldrich
anti-Notch3 ICD	1:2500	Abcam
anti-β-actin	1:5000	Sigma-Aldrich
anti-Calnexin	1:3000	Abcam
anti-Giantin	1:3000	Abcam
anti-LAMP1	1:200	Abcam
anti-EEA1	1:100	Synaptic Systems
anti-V5	1:1000	Life technologies
anti-Ki 67	1:1000	Abcam
anti-Na/K ATPase	1:100	Abcam
anti-PDGFRβ (28E1)	1:1000	Cell Signaling Technology
anti-phospho-PDGFRβ (Y751)	1:1000	Cell Signaling Technology
anti-phospho-PDGFRβ (Y771)	1:1000	Cell Signaling Technology
anti-phospho-PDGFRβ (Y1009)	1:1000	Cell Signaling Technology
anti-phospho-PDGFRβ (Y1021)	1:1000	Cell Signaling Technology
anti-phospho-p42/44 MAPK	1:1000	Cell Signaling Technology
anti-phospho-Akt	1:2000	Cell Signaling Technology
anti-phospho- SHP2	1:1000	Cell Signaling Technology
anti-eIF4E	1:1000	Cell Signaling Technology

Disease Models & Mechanisms • Supplementary information

Supplementary figures

Fig. S1

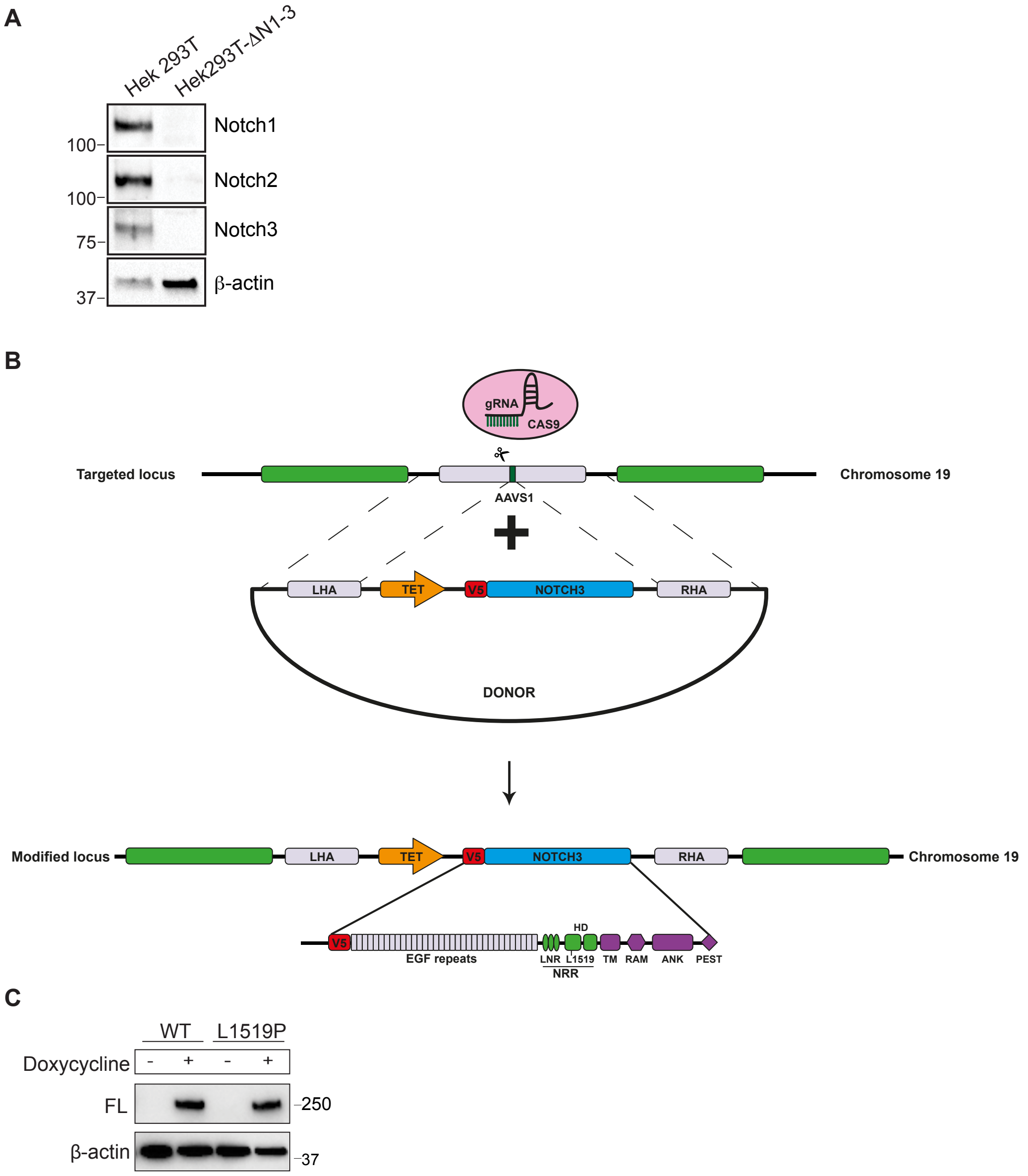

Figure S1. A Western blot of whole cell lysates from HEK 293T ΔN1-3 cells using antibodies for Notch1,2, 3 and β-actin (as loading control). **B** Schematic figure depicting the AAVS1/293T-tet system. **C** Expression of similar levels of wildtype and L1519P NOTCH3 in the HEK293T ΔN1-3 cells in the presence of doxycycline (50ng/ml).

Fig.S2

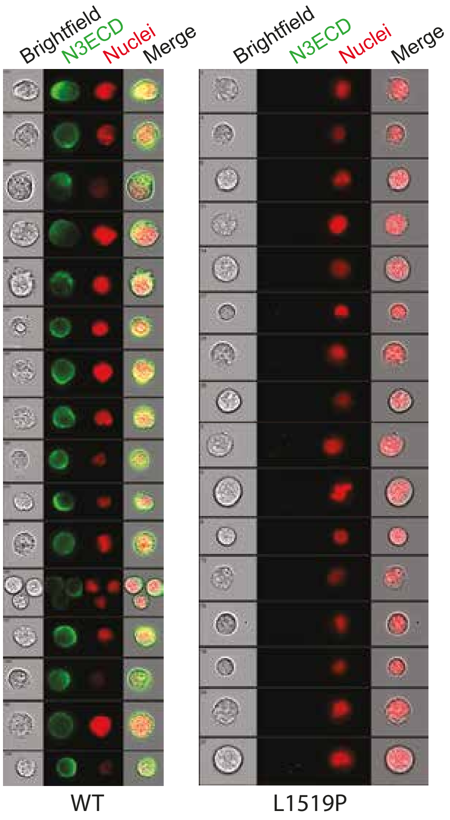

Figure S2. Image stream flow cytometry analysis of NOTCH3 cell surface expression using an antibody to the NOTCH3 extracellular domain (1E4) for wildtype and Notch3^{L1519P} expressing cells.

Disease Models & Mechanisms • Supplementary information

Fig.S3

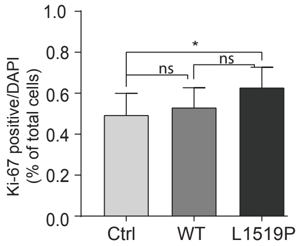

Figure S3. Cell proliferation illustrated as percentage of Ki-67-positive cells normalized to DAPI. Data were obtained from three independent experiments.

Disease Models & Mechanisms • Supplementary information
